# Physiological correlates of ecological divergence along an urbanization gradient: differential tolerance to ammonia among molecular forms of the malaria mosquito *Anopheles gambiae*

**DOI:** 10.1186/1472-6785-13-1

**Published:** 2013-01-07

**Authors:** Billy Tene Fossog, Christophe Antonio-Nkondjio, Pierre Kengne, Flobert Njiokou, Nora J Besansky, Carlo Costantini

**Affiliations:** 1 Institut de Recherche pour le Développement (IRD), Unité Mixte de Recherche MIVEGEC (UM1, UM2, CNRS 5290, IRD 224), Montpellier, France; 2 Laboratoire de Recherche sur le Paludisme, Organisation de Coordination pour la lutte contre les Endémies en Afrique Centrale (OCEAC), Yaounde, Cameroon; 3 Faculty of Sciences, University of Yaounde I, Yaounde, Cameroon; 4 Department of Biological Sciences, Eck Institute for Global Health, University of Notre Dame, Notre Dame, IN, USA

**Keywords:** Local adaptation, Fundamental ecological niche, Environmental stressor, Evolution of tolerance, Urbanization, Malaria, Mosquito

## Abstract

**Background:**

Limitations in the ability of organisms to tolerate environmental stressors affect their fundamental ecological niche and constrain their distribution to specific habitats. Evolution of tolerance, therefore, can engender ecological niche dynamics. Forest populations of the afro-tropical malaria mosquito *Anopheles gambiae* have been shown to adapt to historically unsuitable larval habitats polluted with decaying organic matter that are found in densely populated urban agglomerates of Cameroon. This process has resulted in niche expansion from rural to urban environments that is associated with cryptic speciation and ecological divergence of two evolutionarily significant units within this taxon, the molecular forms M and S, among which reproductive isolation is significant but still incomplete. Habitat segregation between the two forms results in a mosaic distribution of clinally parapatric patches, with the M form predominating in the centre of urban agglomerates and the S form in the surrounding rural localities. We hypothesized that development of tolerance to nitrogenous pollutants derived from the decomposition of organic matter, among which ammonia is the most toxic to aquatic organisms, may affect this pattern of distribution and process of niche expansion by the M form.

**Results:**

Acute toxicity bioassays indicated that populations of the two molecular forms occurring at the extremes of an urbanization gradient in Yaounde, the capital of Cameroon, differed in their response to ammonia. The regression lines best describing the dose-mortality profile differed in the scale of the explanatory variable (ammonia concentration log-transformed for the S form and linear for the M form), and in slope (steeper for the S form and shallower for the M form). These features reflected differences in the frequency distribution of individual tolerance thresholds in the two populations as assessed by *probit* analysis, with the M form exhibiting a greater mean and variance compared to the S form.

**Conclusions:**

In agreement with expectations based on the pattern of habitat partitioning and exposure to ammonia in larval habitats in Yaounde, the M form showed greater tolerance to ammonia compared to the S form. This trait may be part of the physiological machinery allowing forest populations of the M form to colonize polluted larval habitats, which is at the heart of its niche expansion in densely populated human settlements in Cameroon.

## Background

A central theme in evolutionary ecology is understanding the origin and maintenance of adaptations shaped by natural selection. Adaptations result from micro-evolutionary processes occurring within the constraints of the evolutionary dynamics of the fundamental ecological niche, hence understanding such dynamics is crucial to understand the nature and dynamics of adaptations [1]. Physiological limits occur when abiotic conditions become stressful to the extent that organisms fail to survive and reproduce [2], and therefore play a crucial role in determining the fundamental ecological niche and biogeography of organisms (e.g. [3]). In an evolutionary context, abiotic environmental stressors (hereafter, stressors, as a shorthand) constrain the ability of organisms to adapt to environmental changes [4]. Stressors operate not only globally, e.g. in response to climate change across time [5,6] or space (e.g. [7-10]), but also on a local scale in association to geographical heterogeneities (e.g. altitudinal gradients [11,12]), or spatial changes in landscape patterns due to e.g. urbanization or salinisation [13-15]. Accumulation of toxic compounds derived from natural or anthropogenic sources can prevent the occurrence and growth of a species in contaminated areas until the emergence and spread of tolerant genotypes allow colonization of such otherwise unsuitable habitats—a case of ecological niche expansion—which is a phenomenon well known in plants that have adapted to grow in soils contaminated by heavy metals [16].

Populations of the most important malaria vector in tropical Africa, i.e. *Anopheles gambiae sensu stricto*, offer instances of recent niche expansion [17,18] that can provide us with insights about the ecological and molecular mechanisms underlying the emergence and maintenance of adaptations. Niche expansion in *An. gambiae* has resulted in ecological and genetic divergence associated to cryptic speciation of two evolutionarily significant units within this mosquito, named molecular forms M and S, among which reproductive isolation is strong but still incomplete [19,20]. In view of greater genetic similarity of S with the ancestral species of the complex *An. arabiensis*[21-24], it is postulated that the S form is ancestral and the derived M form has emerged through a process of ecological speciation that is still underway [17]. The molecular forms of *An. gambiae* inhabit most eco-climatic domains of their distribution range in West and Central Africa, occurring from xeric steppes and dry savannas at higher latitudes to the Guineo-Congolian rainforest block at lower latitudes [25]. Some ecological divergence, however, is manifested in habitat segregation between the two forms over large geographical extents, with clinal changes in relative abundance resulting in predominance of the M form in marginal environments like coastal and more xeric habitats [17,26-28].

Some evidence suggests that a process of inter-form competition is driving the process of niche expansion [17]. As populations of both M and S are chromosomally [17,25,28,29] and molecularly [23,30,31] distinct between savanna and forest, it is perhaps not surprising that niche expansion of the M form has followed different pathways in these two eco-climatic domains. In the savanna of Burkina Faso and Mali, niche expansion is manifested in the occupation by the M form of habitats [17] and seasons [32,33] of marginal quality, including anthropogenic larval breeding sites of a more stable and complex nature [34], where mosquito predators are more abundant [35]. In the rainforest of Cameroon, the M form has evolved adaptations enabling it to live in urban agglomerates, where its larvae can develop in water collections polluted with decaying organic matter and inorganic waste that occur in slums and other densely populated urban habitats [18]. In Yaounde, where this phenomenon has been observed and described with some degree of detail, niche expansion is manifested in clinal patterns of habitat segregation along urbanization gradients. In this city, it is only the M form that has adapted to breed in polluted anthropogenic water collections. Accordingly, in the forest eco-climatic domain of southern Cameroon this form occurs in the core of urban agglomerates, whereas the S form lives in the surrounding rural settings. The two forms meet and co-exist in sympatry in peri-urban areas where their abundance changes clinally along the urbanization gradient [18].

What factors are responsible for this pattern of habitat segregation underlying niche expansion by forest populations of the M form? Adaptations underlying niche evolution have presumably followed large-scale global phenomena like deforestation and urbanization, which are known to alter profoundly the bionomics and composition of mosquito communities in the tropics [36-38]. Thus, some hints come from observations in Cameroon that the process of niche expansion by *An. gambiae* has produced a novel species association with *Culex quinquefasciatus*, a mosquito pest that can develop in nutrient-rich waters produced by human sewage, such as cesspits or open-air waste drains [39], for which there were no previous historical records in Central Africa [40-42]. We postulated, therefore, that the M form might have developed increased tolerance to those environmental stressors that historically prevented the co-occurrence of *An. gambiae* and *Cx quinquefasciatus* in the same larval habitats. Of manifold environmental stressors occurring in urban aquatic habitats, municipal and domestic waste waters contain large amounts of dissolved nitrogenous pollutants resulting from the decomposition of organic waste matter, industrial processes, agricultural runoff, and sewage effluents, which are more concentrated in *Cx quinquefasciatus* breeding sites [43]. Fertilisers are another source of nitrogenous compounds that can accumulate in residual irrigation water. In Yaounde and Douala, the two major towns of Cameroon where the M form dominates [18], cultivation of market-garden vegetables is common practice in cleared patches interspersed with urban infrastructures, and *An. gambiae* larvae breed in the irrigated furrows of these cultivations. Among nitrogenous compounds, ammonia (in its unionised form NH_3_) is the most toxic to fish and aquatic invertebrates [44,45], and its average concentration is significantly higher in urban *vs.* rural larval habitats where *An. gambiae* pre-imaginal stages are found [46]. Moreover, within the urban habitat, ammonia is more concentrated in polluted as compared to unpolluted breeding sites [47].

In view of the above, we predicted that the M form might have developed increased tolerance to ammonia matching the higher concentration of this compound in its core habitat. To test this expectation, we submitted *An. gambiae* larvae to acute toxicity assays in order to assess the shape and parameters of the functional dose-mortality response under exposure to increasing ammonia concentrations. For this purpose, we tested populations of the M and S forms from localities situated on a transect spanning a gradient of urbanization, where habitat segregation between the two molecular forms was originally recorded [18]. We report the results of these acute toxicity assays and show that the M form, as expected, exhibits greater tolerance to ammonia compared to the S form.

## Methods

### Mosquito populations

**Figure 1:**
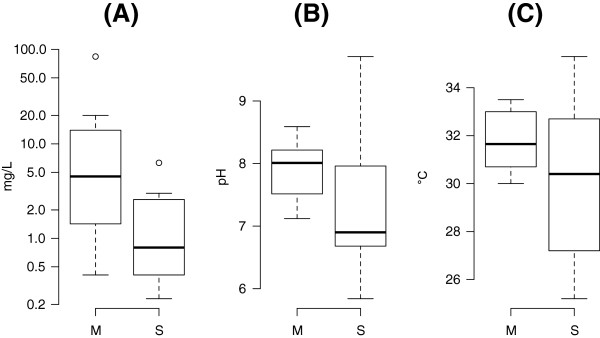
**Summary statistics (median, inter-quartile range, and range) of physico-chemical variables measured in larval breeding sites of *Anopheles gambiae *molecular forms.** S-form sites (*n* = 13) comprise localities 1–5, and M-form sites (*n* = 13, except for ammonia for which *n* = 12) comprise localities 14–16 in Figure 1E of reference [18]. **(A)** Ammonia (mg·L^-1^ total-N); **(B)** pH; **(C)** temperature (°C). It is worth noting that ammonia concentration is presented on the logarithmic scale.

Mosquito larvae were collected at the two extremes of a geographic transect along an urbanization gradient in Yaounde, the capital of Cameroon (localities 1–5 and localities 14–16 shown in Figure 1E of reference [18], where the S and M forms, respectively, largely predominate), and from two additional localities, one in the urban centre (M-site, Voirie Municipale: 3°51’23”N 11°31’00”E), and another at the northern outskirts of Yaounde (S-site, Nkolondom: 3°57’23”N 11°29’18”E), which were selected to increase the sample size and the physiognomical diversity of larval breeding sites. A sub-sample of 254 larvae exposed in the bioassays was molecularly identified to verify that sites were representative of alternative molecular forms, as expected from results of previous surveys [18]. Indeed, molecular identification by a PCR-RFLP protocol [48] confirmed that larvae collected from M and S sites could be considered representative of, respectively, M and S populations. Forty individual breeding sites (21 from M sites, and 19 from S sites) were tested, for a total of 1,917 exposed larvae (1,017 from M sites and 900 from S sites).

### Physico-chemical properties of natural larval habitats

To investigate differences in some physico-chemical properties of natural *An. gambiae* larval habitats, ammonia concentration (mg·L^-1^ total-N), pH, and temperature of the breeding sites from which test larvae were collected were measured using a portable testing kit (CP1000, Wagtech International, Thatcham, Berkshire, UK).

### Bioassay protocol

Ammonia acute toxicity assays followed procedures similar to the standard protocol established by the World Health Organisation for laboratory testing of mosquito larvicidal compounds [49]. Larvae were exposed to nine ammonia concentrations ranging from 5–2000 mg·L^-1^ total-N. Target concentrations were established by serial dilutions in distilled water starting from a commercial 5% ammoniacal solution. Eight to twenty late-stage (III and IV instar) *An. gambiae* larvae from a single breeding site were placed individually in 10-mL test tubes for each of the target concentrations. A control series containing only distilled water was established for each tested breeding site (total number of control larvae across tests: *n* = 412). Larvae were not given access to food to avoid changes in the aquatic milieu. Isolation in individual tubes prevented cannibalism. Initially, larvae were scored at 12, 24, and 36 hours post-treatment, when they were considered dead if they could not dive or emerge at the water surface after touching with the tip of a pipette. Subsequently, for reasons presented below, mortality was scored after 12-hours exposure.

### Statistical analysis

Because of sample size constraints, the total number of exposed and dead larvae from each group of localities (i.e. rural *vs.* urban sites) was pooled and used as the response variable in generalized linear models (GLMs) with a binomial errors structure, while ammonia concentration was fitted as the explanatory variable. Estimation of lethal concentrations (LC) followed a three-pronged approach. First, we explored the dependence of tolerance as a function of duration of exposure to the toxicant. To do so, we estimated the median lethal concentration (LC_50_) at each exposure duration from the parameters of logistic regressions using a *logit* transformation. Because of the presence in some treatment combinations of substantial background mortality (i.e. the natural response occurring even in the absence of, or in addition to the toxicant, as evidenced from mortality in the control batches), we estimated regression parameters by maximum likelihood using custom-written functions in *Mathematica* v.7 (http://www.wolfram.com) following the approach described by Collett ([50], pp. 105–107).

Second, using the data set pertaining to 12-hours exposure we fitted with the statistical software *R* v.2.13.0 (http://www.r-project.org) [51] several competing GLMs characterised by different combinations of link functions and scale of the explanatory variable (cf. Results below). As control mortality at this exposure interval was nil, this time it was not necessary to take into account and correct for the natural response. The minimal adequate models were identified by means of the Akaike information criterion (AIC). Standard errors for lethal concentrations were estimated with the function *dose.p* available in the MASS library.

Third, we used *probit* analysis to estimate the probability density function (PDF) of individual tolerance thresholds to assess their shape, variance, and degree of overlap among the two forms. In toxicity assays, *probit* analysis allows to relate the parameters of the regression line describing the dose-mortality relationship to a Gaussian frequency distribution of tolerance thresholds in the population of individuals tested [50]. Under *probit* analysis, the median lethal concentration expresses the mean (mode), and the inverse of the slope of the regression line expresses the standard deviation of the normal distribution of tolerance thresholds. The ammonia concentrations corresponding to intersections of the PDFs of the M and S forms were estimated with an equation root-finding function in *Mathematica*.

Statistical inference for differences in the physico-chemical variables was performed using non-parametric tests due to heteroscedasticity in these data.

## Results

### Physico-chemical properties of natural larval habitats

The median, inter-quartile range (IQR), and range of ammonia, pH, and temperature measured in *An. gambiae* larval habitats are presented in Figure 1. Ammonia (*P* < 0.02) and pH (*P* < 0.05) were significantly higher in urban M-form compared to rural S-form sites by the Kruskal-Wallis rank sum test. Variation in ammonia concentration was significantly greater (*P* < 0.01) in urban M-form sites by the Fligner-Killeen test. By the same test, temperature showed significantly greater variation (*P* < 0.05) in rural S-form sites (Figure 1).

### Dependence of tolerance on duration of ammonia exposure

**Figure 2:**
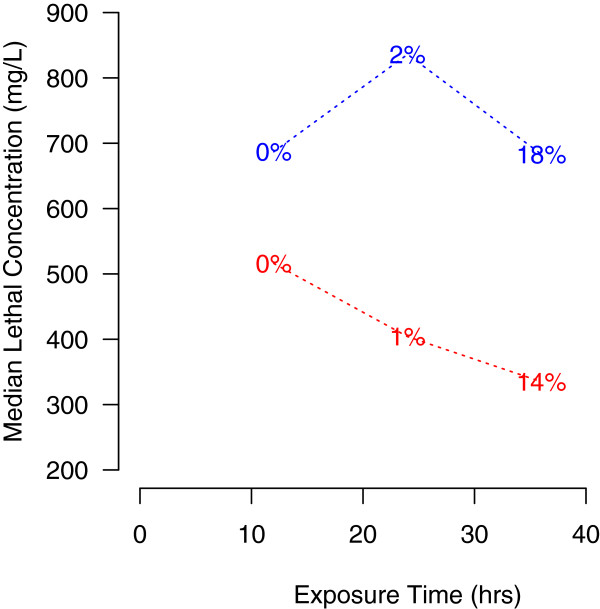
**Dependence of ammonia tolerance, expressed by the median lethal concentration (LC**_**50**_**), on duration of exposure to the toxicant in *Anopheles gambiae *molecular forms (form M: blue; form S: red).** Percent figures represent the larval mortality observed in control batches of each form at different exposure intervals.

The median lethal concentration assessed by the preliminary regression analyses decreased with longer exposure to ammonia in the case of the S form, but not for the M form (Figure 2). Average control mortality at 24 hours (1.3%) was above zero but not significantly different (*P* = 0.25 by Fisher’s exact test) to that recorded 12 hours post-treatment (0%), whereas 36-hours mortality was significantly greater (16.9%) than 24-hours mortality (*P* < 0.0001 by Fisher’s exact test), and exceeded the 5% threshold marking the validity of a toxicity bioassay [49]. Accordingly, we present results based on scoring at 12 hours post-treatment, when control mortality was nil. This time lapse is shorter than that usually adopted by ecotoxicologists to assess ammonia tolerance in aquatic macro-invertebrates [45,52]. However, this interval provides the added benefit of minimizing evaporation, changes in pH (which is known to affect ammonia toxicity and tolerance levels), and changes in the physiological status of larvae due to starvation. Given the increased LC_50_ differential between the M and S forms with longer exposure (Figure 2), scoring mortality after 12 hours provided also a more conservative test for differences between molecular forms in ammonia tolerance.

### Assessment of lethal concentrations

**Figure 3:**
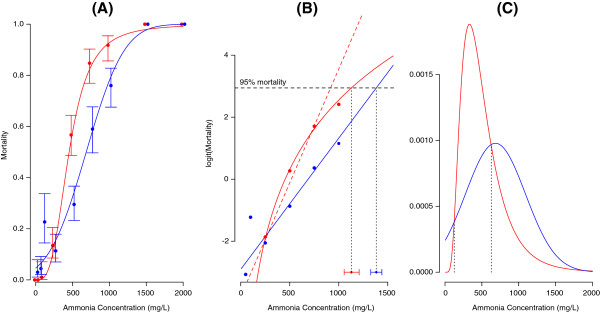
**Acute toxicity of ammonia against larvae of *Anopheles gambiae *molecular forms.(A)** Observed mortality (±95% confidence limits) after 12 hours exposure at increasing concentrations of ammonia (mg·L^-1^ total-N). The points and error bars corresponding to each molecular form are slightly shifted from each other for presentation purposes. Sigmoid curves represent the fitted minimal adequate regression lines plotted on the linear scale (red: S-form sites; blue: M-form sites), from which the lethal concentrations presented in Table 2 were calculated. **(B)** The same data of panel **(A)** drawn on the *logit* scale. The explanatory variable (i.e. ammonia concentration) of the minimal adequate logistic regression model for the S form (red curve) is log-transformed, hence the line plots as a curve because the abscissa on the graph is on the linear scale. The dotted red line represents the alternative model for the S-form data, with ammonia concentration expressed on the linear scale, as in the case of the M form (blue line); this regression line has been used for statistical inference purposes, to compare the functional response of the M form with respect to that of the S form (cf. Results). Diamonds at the bottom right of the scattergram indicate the estimated 95% lethal concentration (LC_95_ ± SE) for M-form sites (blue) and S-form sites (red). Data points for which observed mortality was 0% or 100% are not shown because they map to ± ∞ on the *logit* scale. **(C)** Probability density functions (PDF) of individual tolerance thresholds in populations of the two forms estimated from *probit* analysis (blue: M form, red: S form). Vertical dotted lines identify the ammonia concentration tolerance thresholds where the two PDF curves intersect; above or below these tolerance thresholds, the M population contains a greater proportion of individuals compared to the S form.

Of six competing GLMs fitted to the bioassay data, the one with ammonia concentration on the linear scale and a *probit* link function was minimal adequate for the M form, and that with concentration on the logarithmic scale and a *logit* link function was minimal adequate for the S form (Table 1). Major differences in the model explanatory power were observed mainly depending on the scale of the explanatory variable, i.e. either linear or logarithmic, whereas the differences between models with different link functions were comparatively minor (Table 1). Accordingly, we used the minimal adequate models to estimate ammonia lethal concentrations (Table 2). The fitted curves describing the mortality response to ammonia concentration corresponding to the endpoints presented in Table 2 are shown in Figure 3A. Estimated median and 95% lethal concentrations were higher in the M form compared to the S form. However, mortality was greater in the M compared to the S form at the low end of ammonia concentrations tested.

**Table 1:** **Akaike information criterion (AIC) of competing generalized linear models testing the effect of ammonia concentration on *Anopheles gambiae *molecular forms larval mortality**

**Expl. Variable**	**Molecular Form**					
	**M**			**S**		
	***logit***	***probit***	***log-log***	***logit***	***probit***	***log-log***
CONC.	60.26	**59.23**	59.24	55.71	55.59	107.29
*log*(CONC.)	141.41	166.22	108.86	**27.42**	27.73	33.62

All models have a binomial errors structure but differ in the scale of the explanatory variable (CONC., i.e. ammonia concentration, linear *vs.* log-transformed), or the link function of the linear predictor (*logit vs. probit vs.* complementary log-log). The minimal adequate model for each taxon, i.e. that having the lowest AIC, is shown in bold.

**Table 2:** **Sample estimates of median (LC**_**50**_**) and 95% (LC**_**95**_**) Lethal Concentrations (±95% confidence limits) calculated from minimal adequate generalized linear models assessing the effect of ammonia concentration (expressed in mg·L**^**-1**^**total-N) on *Anopheles gambiae *molecular forms larval mortality**

**Form**	**Lethal Concentration (LC)**			
	**LC**_**50**_		**LC**_**95**_	
M	685	(639 – 729)	1355	(1250 – 1460)
S	453	(421 – 486)	1129	(986 – 1292)

The LC_50_ and LC_95_ presented in the table estimate the concentrations producing 50% and 95% mortality, respectively, in the larval population exposed for 12 hours to ammonia.

### Assessment of differences between forms in the dose-mortality response

To verify whether the dose-mortality response was statistically different between the M and S forms—regardless of link function and scale for the explanatory variable—we compared the logistic regression models with a *logit* link function and ammonia concentration expressed on the same linear scale (Figure 3B), specifying a quasibinomial errors structure due to overdispersion in the data. Although arbitrary, this choice was justified on grounds that the residual deviance was similar in both the M and S forms for models having a *logit* link function and ammonia concentration expressed on the linear scale (Table 1). The relevant statistical test in this case is to verify whether the slope of the two regression lines characterising the dose–response relationship in the M and S forms is significantly different. Indeed, the slope of the regression line was significantly steeper for S sites compared to M sites (likelihood-ratio test: F_1,14_ = 9.13; *P* < 0.01), indicating that the M form expressed higher phenotypic variability in ammonia tolerance compared to the S form (Figure 3B). However, the different slope of the dose–response relationship implies that a comparison of tolerances between the M and S forms depends on the concentration considered. The superior performance of the M form was apparent only at the higher range of concentrations tested. The higher phenotypic variability in ammonia tolerance exhibited by the M population indicates that this form had a larger variance in the distribution of individual tolerance thresholds, i.e. it comprised a greater proportion of individuals with a higher tolerance threshold compared to the S population.

This phenomenon can be seen more explicitly using the results of the GLMs with a *probit* link function. The two Gaussian curves describing the frequency distribution of individual tolerance thresholds for both the M and S forms are plotted in Figure 3C. The two curves, normal for the M form and lognormal for the S form, were parameterized using the results of the minimal adequate GLMs fitted with a *probit* link function. As shown by the two tolerance thresholds where the probability density functions for the M and S forms intersect (dotted lines in Figure 3C), the M population had a greater frequency of individuals with tolerance thresholds ≥625 mg·L^-1^ (~56% versus ~27% in the S form), while the S population had fewer individuals with tolerance thresholds ≤124 mg·L^-1^ (~1% versus ~8% in the M form).

**Figure 4:**
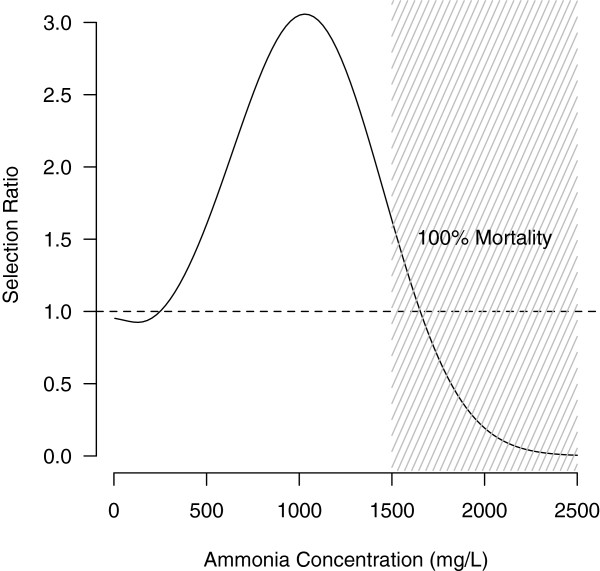
**Estimated relative frequency of M-form *vs. *S-form survivors (Selection Ratio) when culling a mixed M/S larval population using increasing ammonia concentrations.** A Selection Ratio of one (horizontal dashed line) corresponds to equal numbers of M and S survivors. Values above this line identify the region where M-form survivors exceed S-form survivors, and *vice versa* for values below the line. Shading identifies the range of concentrations for which observed larval mortality was 100% in both forms.

These figures help to understand the relative selective effects due to acute ammonia toxicity: a mixed population of 100 larvae of each form exposed to ammonia at 625 mg·L^-1^ is expected to have twice as many M-form survivors (56 *vs.* 27, respectively). Conversely, at 124 mg·L^-1^, the S-form survives slightly better (99 S-form against 92 M-form survivors) but there is a four-fold lower absolute difference (29 *vs.* 7, respectively). These ammonia concentrations correspond to tolerance thresholds for which the difference in survival between the two forms—in favour of the M or the S form, respectively—is maximised (figure not shown). Because the number of survivors is a function of ammonia concentration, however, the *relative* difference in survival between the two forms behaves somewhat differently. Figure 4 shows the ratio of M-form survivors relative to S-form survivors as a function of the culling ammonia concentration: at low concentrations the ratio remains below but close to unity, i.e. the number of S-form survivors only slightly exceeds that of M-form survivors (e.g. at 124 mg·L^-1^, as seen previously, the ratio is ~0.93, which is close to the minimum ratio of 0.924 at 128 mg·L^-1^). Beyond 249 mg·L^-1^, however, the ratio starts to increase steadily reaching a maximum at 1,031 mg·L^-1^, when the number of M-form survivors is three times (3.06-fold) that of S-form survivors (Figure 4). Beyond the maximum, the ratio decreases again towards unity as larval mortality approaches 100% in both forms. Increasing ammonia concentrations, therefore, will disproportionally select for M-form survivors until concentrations are so high that no larva of either form can survive.

## Discussion

In the rainforest of southern Cameroon ecological divergence between two molecular forms of the malaria mosquito *Anopheles gambiae* along urbanization gradients [18] is postulated to be the outcome of niche expansion by the M form, a process driven by disruptive selection and local adaptation to marginal habitats [17]. Historical records indicate that the core of highly urbanized settings constitute an unfavourable habitat for the persistence of *An. gambiae* populations due to the paucity of suitable unpolluted water bodies necessary for the development of its aquatic larvae [41,42,53,54]. In the two major urban agglomerates of Cameroon, Yaounde and Douala [18], and in other large cities lying on the Gulf of Guinea [55-57], larvae of the M form appear to have evolved the ability to survive in polluted breeding sites. The physiological, behavioural, and other life-history traits that are involved in this process are, however, to date largely unknown. Starting from the observation that in the two major urban agglomerates of Cameroon the M form occurs at times in the same breeding sites of *Culex quinquefasciatus*[18], a mosquito pest which is notoriously abundant in waters enriched with nutrients, we postulated that the M form might have developed enhanced tolerance to environmental stressors ensuing from the decomposition of organic matter.

Ammonia is a toxicant resulting from the decomposition of decaying organic matter often contaminating mosquito larval habitats [41,43,46,47,58,59]. Polluting human activities contribute to the generation and accumulation of this contaminant especially in unplanned urban environments in tropical Africa, where accessible water collections containing large amounts of decaying organic matter can be plentiful. Accordingly, by acute toxicity assays we have demonstrated quantitative differences in tolerance to ammonia between the urban population of the M form in Yaounde compared to the parapatric population of the S form occurring in neighbouring rural settings. Higher ammonia tolerance in the M form presumably constitutes an adaptive trait given that this toxicant occurred on average at higher concentration in *An. gambiae* urban larval habitats compared to rural ones. Moreover, urban breeding sites were on average more alkaline than rural ones, a physico-chemical property that enhances ammonia toxicity [45,52]. To confirm the adaptive value of tolerance, however, it will be necessary to demonstrate that individuals with greater ammonia tolerance have a higher relative fitness in the polluted larval habitats of the urban environment compared to individuals with lower tolerance. Additionally, it will be necessary to verify the heritable basis of ammonia tolerance demonstrated in other dipterans [60] to support the notion that disruptive selection has produced the quantitative differences between the M and S forms in this trait. Adaptive phenotypic differentiation can in fact also result from adaptive phenotypic plasticity without underlying genetic differentiation, which is a pre-requisite for local adaptation.

The quantitative differences that we have measured between the M and S forms concern mainly the shape and the variance, more than the mean, of the tolerance response. The M form exhibited a larger variance in the distribution of individual tolerances, whose consequence is that the M population comprised a greater proportion of individuals with a higher tolerance threshold compared to the S population. Under a mode of polygenic inheritance of ammonia tolerance, this pattern can be interpreted to be the outcome of several, not mutually exclusive, processes. First, greater variability in the distribution of thresholds could indicate that the M form may have been subjected to relatively recent disruptive or directional selection for ammonia tolerance, in agreement with results from artificial selection experiments whereby the phenotypic variance of selected lines is generally increased [61]. Second, greater phenotypic variance is expected under selection with assortative mating [62]. If ammonia tolerance is selected along the urbanization gradient according to increasing selection intensities from the rural towards urban localities, on average individuals with greater tolerance will be selected for in the urban environment, and individuals with lower tolerance will be selected for in the rural environment. If mating occurs in the original environment before dispersal, this process will produce some assortative mating correlated to ammonia tolerance. The ensuing gametic-phase disequilibrium in loci influencing ammonia tolerance will result in increased additive genetic variance that will be reflected, if environmental and other genetic components of variance do not decrease, in greater phenotypic variance.

A concurrent interpretation hinges on the homogenising effect of gene flow, which counteracts diversification by selection during local adaptation [63]. Gene flow between the M and S molecular forms is strongly reduced but—apparently—not nil [64-66]. Asymmetric introgression of maladaptive alleles present in the rural S population into the urban M population may hinder greater phenotypic divergence between the two forms, unless the genes underlying this phenotype are linked to genes controlling reproductive isolation between the two forms—a condition that would favour ecological speciation if ammonia tolerance is adaptive. Asymmetric introgression of M-form nuclear genes into the S-form genome (a pattern opposite to that predicted by our observations) has been demonstrated in Guinea Bissau, a relatively restricted geographical area of high hybridization between the two forms [64,65]. In a similar way, neighbouring demes of the larger M-form metapopulation occurring in forested southern Cameroon [18], connected by dispersal to the Yaounde deme, may introduce maladaptive tolerance alleles if these allochthonous demes do not share the same local adaptation mechanisms of the Yaounde deme. Maladaptive alleles would contribute towards greater phenotypic variance in tolerance thresholds observed in the Yaounde M population and could persist in the urban environment because of the presence of breeding sites with lower ammonia concentration, as indicated by the greater variance in ammonia concentration observed in urban compared to rural natural larval habitats.

In the context of recent niche expansion, it is relevant to ask whether the differences in ammonia tolerance observed between the M and S forms from Yaounde predate or are subsequent to the process of ecological divergence and local adaptation to the novel urban habitat. In the absence of historical data it is quite difficult to answer this question. The experimental designs typical of evolutionary toxicology, a discipline that investigates the impacts of environmental pollutants on the genetics of natural populations, include comparison of matched reference and polluted sites [67], which is the approach we have adopted in this study. However, any conclusion derived from this approach is confounded by the ancillary question of whether local adaptation to the urban environment predates or arose subsequent to initial lineage splitting between the molecular forms. Hints to answer these questions are likely to come from studies investigating the nature and degree of tolerance in other M and S geographical populations with varying exposures to naturally occurring pollutants. Despite the limitations of this correlational approach, a match between exposure and degree of tolerance in replicated demes will add credit to the hypothesis that increased tolerance has evolved in response to higher levels of contamination. Further hints will come from a more detailed understanding of the phylogeography of the M and S forms across their distribution range; ultimately, both approaches will shed light on the population structure and history of adaptations in *An. gambiae*.

The difference in ammonia tolerance observed between the M and S forms in Yaounde reflects a process of local adaptation whose generality and limits should be verified in other cities of their distribution range, particularly in regions where populations are akin genetically to those occurring in the rainforest eco-climatic zone of southern Cameroon (e.g. carrying homosequential standard karyotypes like the M and S populations in Yaounde [28]). It is already reported that in some West African cities in the savanna, *An. arabiensis—*another member of the *An. gambiae sensu lato* (*s.l.*) species complex—is extending its distribution southwards into urban environments [68,69], and has developed the ability to breed in larval habitats contaminated by waste waters [70,71]. In East Africa, members of the *An. gambiae s.l.* complex occur in polluted urban larval habitats in Dar-es-Salaam [72], Kisumu, and Malindi [73]. These geographical differences outline the fact that adaptation to urban pollution is happening repeatedly and independently within the species complex, and it is presumably very recent given that urbanization is a relatively novel phenomenon; furthermore, they suggest that lineage splitting between the M and S forms predates adaptation to uban habitats, in agreement with the estimated age of their divergence that is situated well before the Neolithic revolution [74].

### Study caveats

In our acute toxicity bioassays, the difference in tolerance between the two molecular forms appeared only at ammonia concentrations well above those measured in natural larval habitats in and around Yaounde. It must be noted, however, that the potency of a toxicant depends also on the duration of exposure, which in our case was the shortest usually employed by ecotoxicologists when testing ammonia tolerance of macro-invertebrates. Longer exposures usually engender lower lethal concentrations. In the case of the shrimp *Litopenaeus vannamei*, for example, the median lethal concentration of ammonia at 96 hours was approx. 33% of that after 12 hours exposure [75]. From our preliminary analysis, the median lethal concentration after 36 hours exposure in the S form was 65% of that scored at 12 hours, and showed the same kind of decay with time observed in other invertebrates [75]. Nevertheless, we chose to score mortality after 12-hours exposure, and consequently shifted the range of test concentrations towards higher—ecologically less-relevant—values to avoid the influence of external factors like changes in pH or hunger, as evidenced by increased mortality in the control batches.

Ammonia tolerance*,* moreover, might reflect a general physiological response of detoxification and excretion following the same metabolic mechanisms involved in nitrogen homeostasis and osmoregulation [76]. Ammonia is one of several nitrogenous compounds that can be found in nutrient-enriched waters (e.g. those resulting from nitrification of organic matter, such as nitrites and nitrates, or fertilisers, such as urea), whose accumulative toxicity may act additively or synergistically with that due to ammonia [77]. Longer exposure to ammonia and other nitrogenous compounds in natural larval habitats might therefore select for a differential tolerance pattern between the M and S forms akin to that observed in our bioassays in response to short-term exposure to ammonia alone.

If differential tolerance to ammonia contributes to explain habitat segregation of the two molecular forms along the urbanization gradient in Yaounde, however, it is unlikely that it does so solely by selective effects engendered by acute toxicity. Sub-lethal or (sub-)chronic toxicity effects are also likely to operate in this context. It is not yet known, for example, how acute toxicity in *An. gambiae* relates to chronic effects on life history traits affecting fitness. In *Drosophila melanogaster*, fruit-flies artificially selected for ammonia tolerance developed faster and expressed higher viability on ammonia-supplemented media [60]. The aim of this work was to highlight intrinsic physiological differences in tolerance between the M and S forms in their response to toxic levels of ammonia rather than to assess the biopotency of ammonia or look at its chronic sub-lethal effects under longer exposure. This is a matter for future associative and experimental approaches once the ecological processes underlying the cline in the M and S populations abundance along urbanization gradients will be further clarified. Further, it is possible that local adaptation may be associated to differential responses not only to nitrogenous compounds, but also to other environmental stressors characteristic of *An. gambiae* breeding sites [57,72,73,78,79], like temperature, organic and inorganic suspended solids, dissolved substances, emulsions and colloids (including e.g. heavy metals, detergents, oils, or other kinds of nutrients), or biological factors like micro-organisms [80], competitors [81-83], predators [35,84], and their interactions (e.g. [85-87]); these effects need to be investigated at both the population and community level.

In this study we used late-instar larvae collected in the field to test for differential ammonia tolerance between the two molecular forms. Given the spatial segregation of the two forms along the urbanization gradient and the difference in average ammonia concentration between urban *vs.* rural breeding sites, the differences observed might have resulted from prior selection of more tolerant individuals in natural larval habitats before experimental exposure to ammonia in our bioassays. However, if the distribution of tolerance thresholds in the larval population before selection had been the same in both molecular forms, natural selection due to field exposure to greater concentrations of ammonia in the M-form is expected to reduce the variance in tolerance, because only the more tolerant individuals would survive to the third- and fourth-instar stage of development. The fact that we actually observed greater—not reduced—variance in tolerance by the M form is not consistent with this scenario, unless the distributions of ammonia concentrations in urban *vs.* rural larval habitats largely overlapped in the lower range of concentrations, an outcome not manifest in our test samples (cf. Figure 1). Future ammonia tolerance bioassays performed on F2 progeny of field-collected mosquitoes could help resolve this issue and avoid further confounding factors due to maternal effects.

Despite the limitations of this study, it is clear that ecological divergence between the two molecular forms of *An. gambiae* in Yaounde is associated to differences in a physiological response that potentially affect the survival of larvae in natural breeding sites. Development of tolerance may engender costs leading to trade-offs in other fitness-related life-history traits, as observed for East African populations of *An. gambiae* in response to increased tolerance to heavy metals [88]. We speculate, therefore, that development of ammonia tolerance might have fostered niche expansion into the urban environment by forest populations of the M form in Yaounde, while also conferring a competitive disadvantage in the unpolluted larval habitats typical of rural settings in the rainforest block of Cameroon.

## Conclusions

Increased tolerance to pollutants in *An. gambiae s.l.* which is associated with its adaptation to live in densely populated urban environments, bears important potential epidemiological consequences on parasite transmission. If selection for adaptive mechanisms will increase the mean fitness of urban populations, it is conceivable that in the future vector densities and/or survival may increase in densely populated urban environments, a process that would ultimately increase the vectorial capacity of these important malaria vectors. Human population growth projections estimate that, by year 2050, >60% of the population in Western and Central Africa will live in urban areas, against the current ~40% figure and the past ~10% figure in 1950 [89]. Given these emerging trends, therefore, urban transmission is likely to hold an increasingly prominent place in malaria epidemiology, compared to the present situation [90-92]. Moreover, exposure to pollutants and xenobiotics in urban larval habitats may foster the evolution of insecticide resistance [46,47,71,78], thereby compromising our capacity to combat harmful vector-borne diseases like malaria, lymphatic filariasis, or *Anopheles*-transmitted arboviruses. It remains to be seen how the costs associated with increased tolerance may affect other fitness-related traits, e.g. fertility, fecundity or parasite competence. It is only by means of studies investigating the impact of increased tolerance to pollutants on life-history traits affecting fitness that it will be possible to achieve more accurate predictions about the consequences of *An. gambiae s.l.* adaptation to urban environments on vectorial capacity.

## Abbreviations

AIC: Akaike information criterion; F2: Second generation; GLM: Generalized linear model; LC: Lethal concentration; PCR: Polymerase chain reaction; PDF: Probability density function; RFLP: Restriction fragment length polymorphism; sl: Sensu lato.

## Competing interests

The authors declare no competing interests.

## Authors’ contributions

CC, NJB initiated and designed the study. CAN, FN co-ordinated and supervised lab and field work. BTF, CAN performed field work. BTF, PK processed specimens, carried out biological and chemical assays and molecular analyses. CC, BTF carried out statistical analyses and drafted the paper. NJB, CAN, FN, PK critically revised the manuscript. All authors read and approved the final manuscript.
